# Electrocardiographic Assessment of a Continuous Infusion of Lidocaine in Sevoflurane‐Anesthetized Dogs

**DOI:** 10.1155/vmi/1961926

**Published:** 2026-06-12

**Authors:** Ana Paula Martins, Bruna de Moraes Martins Games, Carlos Eduardo de Siqueira, Guilherme Andraus Bispo, Thais Cabral de Oliveira, Élen Almeida Pedreira de Sousa, Juliana Grecco Braz dos Santos, Wagner Luis Ferreira, Paulo Sergio Patto dos Santos

**Affiliations:** ^1^ São Paulo State University (UNESP), School of Veterinary Medicine, Araçatuba, São Paulo, Brazil, unesp.br; ^2^ University of Marilia (UNIMAR), Marilia, São Paulo, Brazil, unimar.br

**Keywords:** cardiovascular monitoring, electrocardiography, lidocaine infusion, sevoflurane

## Abstract

**Introduction:**

Lidocaine is a local anesthetic widely used in veterinary medicine and can be administered intravenously as a continuous infusion during general anesthesia. In addition to its analgesic and anti‐inflammatory properties, lidocaine has antiarrhythmic effects that may influence cardiac electrical activity during anesthetic procedures.

**Objective:**

To evaluate whether continuous intravenous infusion of lidocaine in dogs anesthetized with sevoflurane alters electrocardiographic and hemodynamic variables.

**Study Design:**

Prospective clinical study.

**Animals:**

Twenty healthy dogs.

**Methods:**

Dogs were anesthetized with sevoflurane administered via face mask, followed by orotracheal intubation and anesthetic maintenance with the same agent. After stabilization of the anesthetic plane, baseline measurements were recorded. The animals were randomly allocated into two groups: control group (CG) and lidocaine group (LG). In the LG, lidocaine was administered as an intravenous bolus (2 mg/kg) followed by continuous infusion at a rate of 100 μg/kg/min. The CG received 0.9% saline solution at equivalent volumes and infusion rates. Evaluations were performed at baseline and at 20, 40, 60, and 80 min after the start of the infusion. The variables analyzed included heart rate (HR), systolic, mean, and diastolic arterial blood pressures (SBP, MAP, and DBP, respectively), and electrocardiographic variables such as P wave duration, PR interval, QRS complex duration, QT interval, and P wave and R wave amplitudes.

**Results:**

No significant differences were observed between groups or across time points for any of the evaluated variables.

**Conclusion and Clinical Relevance:**

Continuous intravenous infusion of lidocaine at the evaluated dose did not produce significant electrocardiographic or hemodynamic alterations in dogs anesthetized with sevoflurane under the conditions of this study, suggesting no detectable impact on cardiovascular function in this experimental setting.

## 1. Introduction

Inhalational anesthesia is widely used in veterinary medicine for the induction and maintenance of general anesthesia in dogs. However, when administered as the sole anesthetic technique, inhalant anesthetics may require relatively high concentrations to maintain an adequate anesthetic plane, which may result in cardiovascular and respiratory depression [1]. In this context, balanced anesthesia, characterized by the combination of different drugs with complementary mechanisms of action, has been increasingly used to reduce the dose of inhalational anesthetics and minimize their adverse effects [2].

Lidocaine is a local anesthetic widely used in both human and veterinary medicine and can be administered through different routes. In addition to its local anesthetic properties, lidocaine has analgesic, anti‐inflammatory, and antiarrhythmic effects [3]. In recent years, continuous intravenous infusion of lidocaine has gained increasing attention as part of multimodal anesthetic protocols [4, 5].

Previous studies have demonstrated that the administration of lidocaine may contribute to the reduction of the minimum alveolar concentration (MAC) of inhalational anesthetics when used as an adjuvant drug [6]. This characteristic may allow lower concentrations of inhalational anesthetics to be used during anesthesia, potentially contributing to greater cardiovascular stability.

In the cardiovascular system, lidocaine acts as a Class IB antiarrhythmic agent and is commonly used for the treatment of ventricular arrhythmias. Its mechanism of action involves the blockade of sodium channels, leading to stabilization of cardiac electrical activity and reduction of abnormal impulse conduction. These effects may be particularly relevant during anesthesia, since inhalational anesthetics can influence cardiovascular function and may increase myocardial sensitivity to catecholamines [7].

Electrocardiography is an important diagnostic method used to evaluate the electrical activity of the heart and to detect arrhythmias and conduction disturbances [8]. During anesthesia, electrocardiographic monitoring allows the assessment of potential drug‐induced alterations in cardiac rhythm and conduction [9].

Considering the increasing use of lidocaine as a continuous intravenous infusion in veterinary anesthesia, it is important to investigate its potential effects on electrocardiographic variables during anesthetic procedures. Therefore, the aim of the present study was to evaluate the electrocardiographic variables of dogs anesthetized with sevoflurane undergoing continuous lidocaine infusion.

## 2. Materials and Methods

### 2.1. Animals

The study was approved by the local university ethics committee (CEUA, no. 564/2020). The experiment was conducted at the Laboratory of Anesthesia and Experimental Surgery at the São Paulo State University (UNESP), School of Veterinary Medicine of Araçatuba (FMVA).

The study included twenty dogs of different breeds, males and females, aged between one and 5 years and weighing between 4 and 20 kg. All animals were scheduled to undergo elective sterilization procedures (ovariohysterectomy or orchiectomy), which were performed after the experimental period.

Inclusion criteria required that the dogs be considered healthy, classified as ASA I (American Society of Anesthesiologists), based on physical examination and complementary laboratory tests. These tests included complete blood count, serum biochemical profile, and serology for leishmaniasis. In addition, participation in the study required the signing of an Informed Consent Form (ICF) by the animals’ owners.

Dogs were excluded if abnormalities were detected during the clinical examination or laboratory tests, or if serology for leishmaniasis was positive.

The animals were obtained from a neutering campaign and were included in the study prior to the surgical procedures.

### 2.2. Experimental Protocol

The dogs underwent an 8‐hour food fast without water restriction before the experimental procedure. The animals were weighed, and trichotomy was performed in the region of the dorsal metatarsal artery for invasive blood pressure monitoring and in the region of the right cephalic vein for placement of an intravenous catheter for administration of the experimental solutions.

After venous access was obtained, the animals were kept at rest for approximately 20 min in an environment with controlled temperature (approximately 23°C) to reduce stress.

The animals were randomly allocated into two experimental groups: control group (CG) and lidocaine group (LG). The experimental treatments were prepared and administered by an investigator not involved in data collection. Evaluators responsible for data collection and analysis were blinded to group allocation. However, the investigator responsible for treatment preparation and administration was not blinded due to the nature of the procedure. Randomization was performed using an online randomization generator.

No premedication or additional drugs were administered prior to anesthetic induction. Anesthetic induction was performed with sevoflurane at a concentration of 5% in 100% oxygen administered by face mask. After loss of the laryngotracheal reflex, the animals were intubated and connected to an anesthetic circuit, and anesthesia was maintained using sevoflurane. Animals were allowed to breathe spontaneously throughout the experimental period, and end‐tidal CO_2_ (ETCO_2_) was continuously monitored to ensure adequate ventilation. End‐tidal sevoflurane concentration was continuously monitored using a multiparametric monitor and maintained at a constant level adequate to sustain a stable anesthetic plane throughout the experimental period. The inspired and end‐tidal sevoflurane concentrations were maintained constant throughout all evaluation time points, with adjustments made only when necessary to preserve a consistent depth of anesthesia among animals. Fluid therapy was maintained using an isotonic crystalloid solution (lactated Ringer’s solution) administered at a constant rate of 5 mL/kg/h throughout the anesthetic period.

After stabilization of the anesthetic plane for 15 min, baseline measurements were recorded before administration of the experimental solutions, and this moment was designated as *T*_0_. In the LG, an intravenous bolus of lidocaine (2 mg/kg) was administered, followed by continuous rate infusion (CRI) at 100 μg/kg/min for a total duration of 80 min, corresponding to the experimental time points (*T*_0_ to *T*_80_). The CRI was discontinued immediately after completion of the final evaluation (*T*_80_), prior to any surgical procedure. In the CG, 0.9% saline solution was administered at equivalent volumes and infusion rates. Measurements were recorded at baseline and at 20, 40, 60, and 80 min after the start of the infusion (*T*_0_, *T*_20_, *T*_40_, *T*_60_, and *T*_80_, respectively).

### 2.3. Electrocardiographic and Hemodynamic Evaluation

Electrocardiographic recordings were obtained using a computerized electrocardiograph (ECGPC TEB, Tecnologia Eletrônica Brasileira, São Paulo, Brazil) with a speed of 50 mm/s and standard calibration of 10 mm = 1 mV, registering the standard limb leads DI, DII, DIII, aVR, aVL, and aVF. All electrocardiographic measurements were performed using lead II. Electrocardiographic measurements were calculated as the mean of three consecutive cardiac cycles. All electrocardiographic measurements were performed by a single trained evaluator who was blinded to group allocation.

The electrocardiographic variables evaluated included P wave duration, PR interval, QRS complex duration, QT interval, and P wave and R wave amplitudes. Heart rate (HR) and cardiac rhythm were also assessed.

Hemodynamic variables included HR and systolic, mean, and diastolic arterial pressures. Blood pressure measurements were obtained through invasive monitoring by catheterization of the dorsal metatarsal artery connected to a multiparametric monitor (Cardiocap/5—GE Healthcare Clinical Systems Equipamentos Médicos Ltda, São Paulo, SP). The pressure transducer was zeroed at the level of the right atrium prior to data collection. Adequate waveform quality was confirmed prior to and during data collection, based on the presence of a characteristic arterial waveform with a rapid upstroke, clear systolic peak, and visible dicrotic notch, without evidence of damping or artifacts.

### 2.4. Statistical Analysis

Data normality was assessed using the Shapiro–Wilk test. The repeated‐measures design allowed each animal to serve as its own control over time, accounting for intraindividual variability and the correlation between sequential measurements. Variables with normal distribution were analyzed using two‐way repeated measures analysis of variance (ANOVA), considering the effects of group, time, and their interaction. When significant effects were detected, Tukey’s post hoc test was applied. For variables that did not meet normality assumptions, appropriate nonparametric tests for repeated measures were used.

A significance level of *p* < 0.05 was adopted.

## 3. Results

The hemodynamic variables HR, systolic blood pressure (SBP), mean arterial pressure (MAP), and diastolic blood pressure (DBP) are presented in Table 1. No statistically significant differences were observed between the CG and the LG at the evaluated moments. Likewise, no significant differences were observed among the experimental moments within each group.

**TABLE 1:** Hemodynamic variables in dogs anesthetized with sevoflurane receiving lidocaine infusion (LG) or saline (CG).

**Hemodynamic variables**					
		**HR**	**SBP**	**DBP**	**MAP**
Groups	Moments	$\overset{¯}{\text{x}}$ ± *s*	$\overset{¯}{\text{x}}$ ± *s*	$\overset{¯}{\text{x}}$ ± *s*	$\overset{¯}{\text{x}}$ ± *s*
LG	*T*_0_	118 ± 21	105 ± 14	66 ± 08	79 ± 09
	*T*_20_	110 ± 12	101 ± 13	67 ± 11	78 ± 11
	*T*_40_	109 ± 12	104 ± 12	66 ± 09	78 ± 09
	*T*_60_	108 ± 16	98 ± 10	65 ± 11	77 ± 10
	*T*_80_	116 ± 12	104 ± 13	71 ± 15	83 ± 15

CG	*T*_0_	122 ± 13	104 ± 09	71 ± 08	83 ± 08
	*T*_20_	111 ± 12	102 ± 08	67 ± 10	79 ± 08
	*T*_40_	111 ± 10	104 ± 08	70 ± 11	80 ± 08
	*T*_60_	109 ± 15	101 ± 11	67 ± 08	78 ± 07
	*T*_80_	111 ± 12	105 ± 11	69 ± 11	81 ± 11

*Note:* Values are expressed as mean ± SD. No significant differences were detected between groups or among time points (*p* > 0.05).

The electrocardiographic variables related to wave and interval duration, including P wave duration, PR interval, QRS complex duration, and QT interval, are presented in Table 2. No statistically significant differences were observed between groups or among the evaluated moments.

**TABLE 2:** Duration (in milliseconds) of the *P* wave, PR interval, QRS complex, and QT interval in dogs anesthetized with sevoflurane receiving lidocaine infusion (LG) or saline (CG).

**Electrocardiographic variables (duration)**					
		***P* wave**	**PR interval**	**QRS complex**	**QT interval**
Groups	Moments	$\overset{¯}{\text{x}}$ ± *s*	$\overset{¯}{\text{x}}$ ± *s*	$\overset{¯}{\text{x}}$ ± *s*	$\overset{¯}{\text{x}}$ ± *s*
LG	*T*_0_	50 ± 3	97 ± 14	64 ± 4	229 ± 16
	*T*_20_	50 ± 3	99 ± 13	62 ± 5	225 ± 15
	*T*_40_	50 ± 4	98 ± 13	63 ± 6	224 ± 17
	*T*_60_	50 ± 4	98 ± 12	64 ± 5	229 ± 16
	*T*_80_	53 ± 5	95 ± 15	66 ± 5	224 ± 17

CG	*T*_0_	48 ± 3	90 ± 13	62 ± 5	227 ± 16
	*T*_20_	49 ± 3	94 ± 12	63 ± 4	229 ± 18
	*T*_40_	50 ± 5	92 ± 13	67 ± 4	233 ± 18
	*T*_60_	50 ± 4	95 ± 12	65 ± 4	236 ± 16
	*T*_80_	53 ± 5	96 ± 14	67 ± 4	236 ± 16

*Note:* Values are expressed as mean ± SD. No significant differences were detected between groups or among time points (*p* > 0.05).

The P wave and R wave amplitudes are presented in Table 3. No statistically significant differences were observed between groups or among the evaluated moments.

**TABLE 3:** Amplitudes (in millivolts) of the *P* wave and *R* wave in dogs anesthetized with sevoflurane receiving lidocaine infusion (LG) or saline (CG).

**Electrocardiographic variables (amplitudes)**			
		***P* wave**	***R* wave**
Groups	Moments	$\overset{¯}{\text{x}}$ ± *s*	$\overset{¯}{\text{x}}$ ± *s*
LG	*T*_0_	0.25 ± 0.11	1.29 ± 0.45
	*T*_20_	0.23 ± 0.11	1.26 ± 0.42
	*T*_40_	0.23 ± 0.12	1.33 ± 0.44
	*T*_60_	0.24 ± 0.12	1.29 ± 0.44
	*T*_80_	0.26 ± 0.10	1.36 ± 0.37

CG	*T*_0_	0.21 ± 0.08	1.22 ± 0.38
	*T*_20_	0.21 ± 0.07	1.37 ± 0.42
	*T*_40_	0.20 ± 0.08	1.37 ± 0.44
	*T*_60_	0.20 ± 0.07	1.36 ± 0.49
	*T*_80_	0.22 ± 0.07	1.39 ± 0.39

*Note:* Values are expressed as mean ± SD. No significant differences were detected between groups or among time points (*p* > 0.05).

## 4. Discussion

HR did not show significant differences between moments within each group or between groups and remained within physiological limits for the species in both groups. Sevoflurane presents greater cardiac stability compared with other inhalational agents such as isoflurane [10]. However, dogs anesthetized with sevoflurane may show higher HR values when compared with isoflurane at equipotent doses, and anesthetic induction performed only with sevoflurane may result in HR values above baseline levels [11]. The use of sevoflurane without opioid premedication may maintain HR stability [10]. In the present study, anesthetic induction was performed using sevoflurane without opioid premedication, and no relevant changes in HR were observed. The findings of the present study are consistent with previous reports indicating minimal cardiovascular effects of lidocaine when administered as a continuous intravenous infusion in anesthetized dogs. Several studies have demonstrated that lidocaine, at clinically relevant doses, does not significantly alter HR, supporting its use as part of balanced anesthetic protocols [6, 12, 13].

Systolic, mean, and diastolic arterial pressures did not show significant differences between moments or between groups and remained within physiological limits for the species. These findings are consistent with those reported in dogs anesthetized with isoflurane receiving continuous lidocaine infusion [2]. However, some discrepancies have been reported in the literature, particularly regarding mild reductions in blood pressure. These differences may be attributed to variations in anesthetic protocols, including the use of premedication, differences in inhalant anesthetic concentrations, or variations in lidocaine dosing regimens. Lidocaine administered at high doses may increase arterial pressure due to reduction in inhaled anesthetic requirements [12, 13]. In the present study, MAP remained stable throughout the anesthetic procedure, suggesting minimal influence of lidocaine infusion at the evaluated dose.

It is important to note that all measurements were obtained prior to the surgical procedure, with animals maintained under stable general anesthesia and without surgical stimulation. Therefore, variations in cardiovascular parameters and sevoflurane requirements were not influenced by nociceptive surgical stimuli, allowing a more direct assessment of the effects of lidocaine infusion under controlled anesthetic conditions.

Regarding electrocardiographic variables, continuous lidocaine infusion did not produce rhythm disturbances, conduction abnormalities, or beats of nonsinus origin. Cardiac rhythm was considered sinus due to the presence of P waves in all tracings. Electrocardiographic parameters were interpreted based on reference values described for dogs [14].

The duration and amplitude of the P wave, related to atrial electrical conduction, remained stable without significant differences between moments or groups. The same pattern was observed for the PR interval, indicating that continuous lidocaine infusion associated with sevoflurane did not significantly influence atrioventricular conduction. In addition, the mean values obtained were close to those described for the canine species [14].

Ventricular depolarization, represented by the QRS complex, also showed no significant differences between moments or between animals that received saline or lidocaine. Previous research reported differences in QRS duration when lidocaine was associated with dexmedetomidine, suggesting that lidocaine may not attenuate second‐degree atrioventricular block caused by dexmedetomidine at higher doses [15]. In the present study, the stability of this variable suggests that lidocaine infusion did not alter ventricular myocardial electrical conduction.

The R wave amplitude, which reflects ventricular contractile strength [16], remained within normal limits and did not differ between groups or experimental moments.

No significant differences were observed in the QT interval between groups or across experimental moments. The QT interval represents ventricular systole and is inversely proportional to HR [16], being commonly used to evaluate the effects of drugs on cardiac dynamics [17].

This study has some limitations that should be considered when interpreting the results. Although the study included dogs of different breeds, sexes, and body weights, all animals were clinically healthy (ASA I), which likely reduced physiological variability. However, this heterogeneity may still have influenced the results and was not included as a factor in the statistical analysis. The relatively small sample size, defined based on previous studies with similar experimental designs and ethical considerations regarding animal use, may have limited the ability to detect subtle but clinically relevant differences between groups. A formal a priori power analysis was not performed, which should be considered when interpreting the absence of statistically significant differences, as the possibility of a Type II error cannot be excluded.

In addition, the inclusion of only healthy animals may restrict the extrapolation of the findings to dogs with systemic disease or altered physiological status. Furthermore, the monitoring period was relatively short, and therefore, potential delayed or cumulative effects of lidocaine infusion may not have been detected. Despite the use of standardized experimental conditions to minimize variability, these factors should be taken into account when interpreting the results and designing future studies. On the other hand, the inclusion of a heterogeneous population may enhance the external validity of the findings, reflecting clinical conditions more closely.

## 5. Conclusions

Continuous lidocaine infusion in dogs anesthetized with sevoflurane did not result in significant hemodynamic or electrocardiographic changes under the conditions of this study. HR, arterial pressure, and cardiac conduction parameters remained within physiological limits throughout the experimental period. These findings suggest that lidocaine infusion at the evaluated dose was not associated with detectable alterations in cardiovascular function in healthy dogs anesthetized with sevoflurane.

## Funding

This research was supported by the São Paulo Research Foundation (Fundação de Amparo à Pesquisa do Estado de São Paulo—FAPESP) (2020/08009‐0).

## Conflicts of Interest

The authors declare no conflicts of interest.

## Data Availability

The data used to support the findings of this study are available from the corresponding author upon reasonable request.
